# Intrinsic CanMEDS Competencies Expected of Medical Students During Emergency Medicine Core Rotation: A Needs Assessment

**DOI:** 10.7759/cureus.3316

**Published:** 2018-09-17

**Authors:** Tahsin Khan, Teresa M Chan

**Affiliations:** 1 Family Medicine, Kingston General Hospital and Hotel Dieu Hospital, Queens University, Kingston, CAN; 2 Health Sciences, McMaster University, Hamilton, CAN

**Keywords:** needs assessment, clerkship, medical student, free open access medical education, foam

## Abstract

Objectives

There are few high-quality free open-access medical (FOAM) education resources to guide medical students in the development of key non-medical expert skills and competencies during their emergency medicine (EM) clerkship core rotation. In our endeavor to develop a novel online educational EM curriculum for medical students, a needs assessment is required to effectively address needs specifically focused on aptitudes that are deemed to be imperative by educators in the EM academia.

Methods

An online needs assessment survey was developed and shared with residents, staff, nurses, and program/clerkship directors of Canadian emergency medicine programs by email correspondence and embedding the form on CanadiEM.org. The survey consisted of twelve proposed topics for a potential EM curriculum, which were graded on a five-point Likert scale. Free-typed responses for additional topics were also solicited from participants.

Results

Over the course of four weeks, 84 participants responded to the survey. Participants outside of North American were excluded (n=10). Most participants were North American staff physicians (n=52), which included residency program directors (n=10) and clerkship directors (n=6), followed by residents (n=14), and nurses (n=8). All 12 topics proposed by the authors were considered important for inclusion in an EM curriculum. Nine additional topics were identified from typed free-text responses. Top ranking topics included: how to present a case to an EM staff or resident, how to chart patient encounters, and how to effectively communicate with nurses and other healthcare professionals.

Conclusions

This online needs assessment analysis revealed a total of 21 topics that were deemed to be relevant to the development of an online curriculum to foster the development of core competencies of medical students during their EM core rotation.

## Introduction

Within emergency medicine (EM), there has been enormous growth in online educational resources (OERs), which includes free open-access medical education (FOAM) resources. Presently, there are abundant FOAM content that guide learners to develop approaches to common clinical presentations and differential diagnoses [1-8]. In addition to common clinical presentations, these resources mainly focus on procedural skills, ECG interpretation, and resuscitation [9].

We have observed a lack of high-quality FOAM resources to guide medical students in the development of skills necessary for effective communication and documentation, which are components of the CanMEDS intrinsic role competencies in Canada [10]. Colloquially, these are often termed “soft skills” by students and educators. For example, key skills such as referring patients to consultants (collaboration), managing of “difficult” patients (communication), and even the process of charting in the emergency department (ED) (professional, collaborator), are rarely featured in FOAM resources. Since the quality and consistency of teaching through experiential learning (i.e. bedside) can be highly variable depending on a student’s supervising physician, it is not feasible to expect them to teach these skills repeatedly with the same level of regularity as new groups of medical students rotate through the ED every four to eight weeks.

During their training, medical students are expected to develop a wide range of competencies that are outlined by CanMEDS and Accreditation Council for Graduate Medical Education (ACGME) [10,11]. We attempted to uncover needs that EM educators in North America perceived to be important for a CanMEDS-based FOAM curriculum tailored for medical students in EM core rotation, with emphasis on effective communication and documentation. This needs assessment analysis will not only be useful for clerkship directors who directly influence curriculum development, but will also be useful for all EM educators, who interact with medical students in the ED on a regular basis. Ultimately, we plan to create novel OERs, or supplement gaps in existing ones, to deliver a well-rounded online curriculum that will aid EM educators in the teaching of communication and documentation skills to medical students.

## Materials and methods

Materials

We received an exemption from our institutional review board (the Hamilton Integrated Research Ethics Board). In Canada, the Tri-Council policy statements specify that program development is exempt from research. As such, since this was educational needs assessments (exploring what the needs were for the intention of developing a blog series) and quality improvement (for the blog) are exempt. 

The needs assessment was developed on Google Forms (Mountainview, CA, USA). The survey was piloted on a few non-participatory EM clinician educators, and feedback was incorporated.

We collected basic demographic information from solicited practitioners, such as professional role (e.g. nurse, resident, staff, program director, etc.), country of medical practice, and practice setting (academic/tertiary hospital, rural community hospital, urban community hospital, etc.).

The survey consisted of a series of 15 questions. Twelve questions represented a topic within the domain of EM clerkship that were deemed to be relevant by the investigators, which align to the Canadian EM entrustable professional activities and the ACGME EM competencies. On a Likert scale, participants were asked to rate the importance of each topic in the context of EM core clerkship competencies (Table 1). The scale consisted of five selections: strongly disagree, disagree, neutral, agree, strongly agree. Prior to prompting participants’ opinions on the relevance of each of these topics, they were asked for a free-typed text response to the following question: “What are some common teaching points that clinical clerks in the ED need to be taught?”. The question was phrased as such, rather than explicitly asking about teaching points on “soft skills”, because the definition of what constitutes these skills may vary significantly from one educator to another, and not all educators are equally familiar with CanMEDS roles. In addition, by phrasing question broadly in a manner that did not exclude topics pertinent only to “soft skills” teaching topics, we were able to elicit input on other practical skills that educators deem important, but are not necessarily effectively taught within medical school curricula (e.g. point-of-care ultrasound skills, attaching ECG leads, etc.) At the end of the survey, participants were solicited to respond to two queries: “Please enter comments (if any) on any of the aforementioned topics", and “Please describe other topics that you consider to be pertinent”.

**Table 1:** Rankings of proposed curriculum topics *Multiple topics with the same ranking

Rank	Average Likert Score (Min = -2, Max = +2)	Topic
1	1.53	How to present a case to an EM staff or resident
2	1.33	How to chart patient encounters in EM
3	1.18	How to communicate with nurses (e.g. appropriateness of requests, questions)
4	1.16	How to reassess patients effectively? What are the key elements involved?
5*	1.10	How to generate effective management and disposition plans for patients?
5*	1.10	How to deal with “difficult” patients, including but not limited to patients that exhibit: lack of cooperation, frustration with wait times, refusal to answer questions, use of profanity)
6	1.03	How to write an ER procedure note (e.g. laceration repair, casting, incision and drainage)
7	1.01	A practical framework for requesting consultations in the ER
9	1.00	Roles and expectations of a medical student during traumas and resuscitations
10	0.96	Key elements involved in safe and effective handovers
11	0.90	A practical framework for breaking bad news
12	0.66	Time management during an ER shift

Population

The form was embedded on the CanadiEM site (canadiem.org), inviting emergency physicians, residents and nurses to respond via this mechanism. The link to the form was distributed to all the EM program directors (n=17) and clerkship directors in Canada (n=17). These individuals were allowed to invite other study participants via the link to the survey. In addition, the survey was posted on Canadiem.org, so that followers of the website would also be able to participate. Students and non-EM practitioners were excluded from the study as outliers. We utilized a convenience sampling approach with a four-week window for survey responses. One reminder was sent to the aforementioned study participants one week prior to the deadline. 

Data analysis

Responses from three clinical clerks and one non-EM physician were discarded because these individuals either did not exemplify the role of an EM educator and would not have been in a position to reliably assess the competency of EM clerks. Responses of eight physicians who practice medicine outside of North America were excluded due to noteworthy differences in their medical education system that may not be representative. American EM physicians were included since many Canadian students engage in electives within the USA. Participant demographics were analyzed using descriptive statistics using Microsoft Excel 2017. After discarding outliers, a total of 74 responses were used for study analysis. Each item on the Likert scale was assigned a single iterative integer value of -2 to +2, from “strongly disagree” to “strongly agree”, respectively. “Neutral” options were assigned a score of zero. For each topic, the total sum of integer values was calculated across all responses and subsequently divided by the total number of responses to determine the mean score of the respective topic. Prior to the study, an a priori threshold was established arbitrarily based upon consensus by both investigators (K = 1.0). A score above zero would have deemed the respective topic(s) to be relevant to the development of an online educational resource in the future. A score below zero would have deemed them to be low-yield and relatively unimportant. The same methodology was applied to exclusively analyze the responses of each healthcare profession (e.g. nurses, residents, staff and program/clerkship directors) independently. A thematic analysis of all written responses suggesting additional topics were analyzed by both investigators (TK, TC) and agreed upon by consensus (K = 0.96).

## Results

Demographics

Over a period of four weeks, from March 21, 2017 to April 21, 2017, 84 participants completed the needs assessment survey, consisting of EM staff physicians (n = 52, 46.4%), residents (n=14, 17.9%), program directors of EM residency programs (n = 10) (13.1% of survey population, but 71.4% of PDs nationwide), clerkship directors of EM clerkship programs (n=6) (7.1% of survey population), and nurses (n=8, 10.7%). Most of the respondents worked in Canada (53.3%) and United States (34.7%) in academic tertiary care centers, where most medical students acquire training. The remainder (n=10), which were excluded, were from the United Kingdom, New Zealand, Spain, India, Germany, and Australia.

Quantitative analysis

Considering that all proposed topics received an average Likert score greater than zero, all proposed topics were deemed to have greater than neutral importance based on the responses of all participants (Table 1). Independent analysis of each health care profession’s responses (e.g. nurses, residents, staff and directors) revealed the same result.

Qualitative analysis

**Figure 1:**
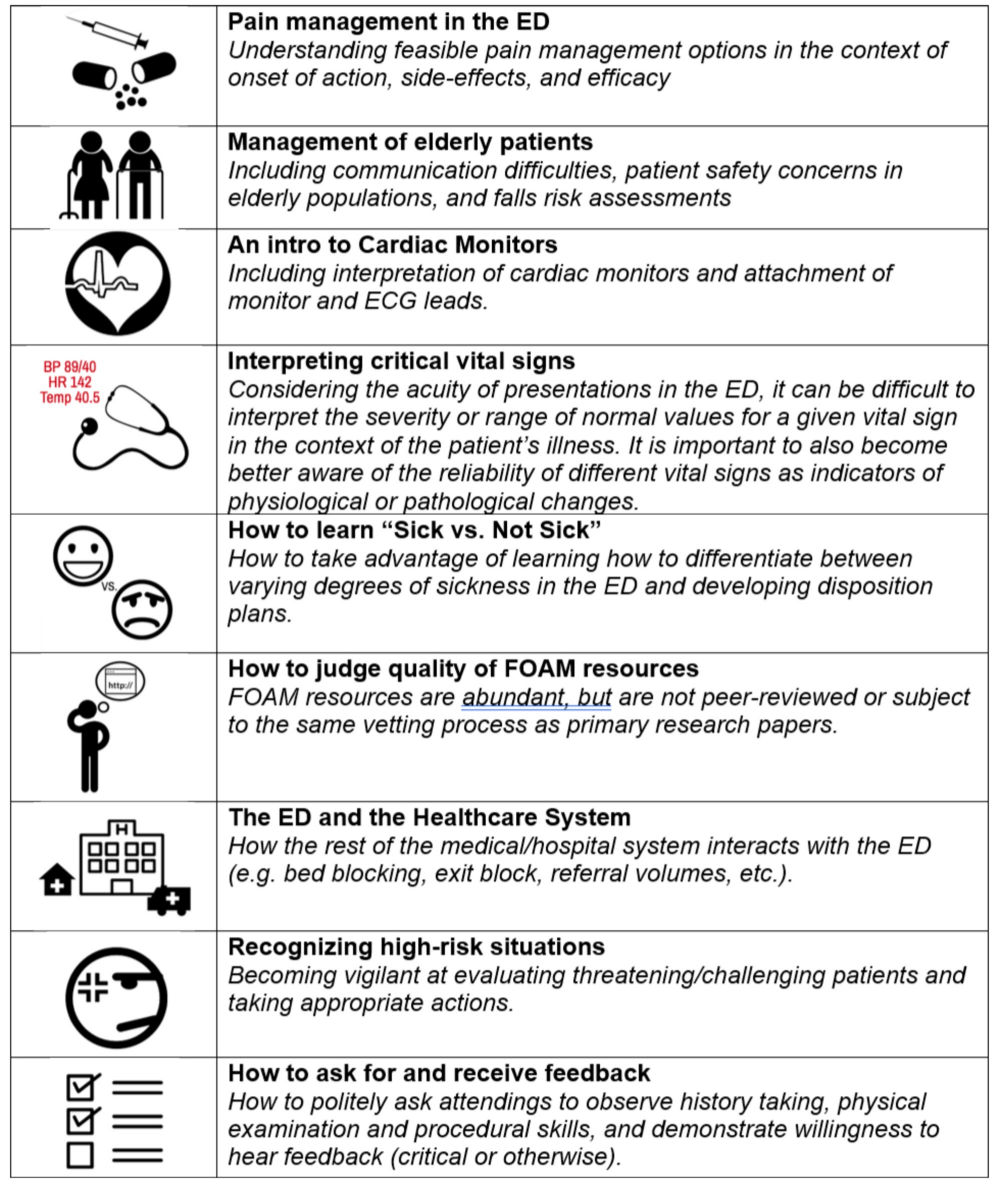
Topics suggested via qualitative comments

Analysis of common themes from additional topics proposed by participants in free-type text format revealed nine unique topics that were not previously considered by the investigators. These topics were summarized and have been outlined in Figure 1.

Based on the suggestions for additional topics proposed in the free-typed text, participants emphasized the importance of improving physical exam and procedural skills by receiving feedback based on direct observation by the supervising physician. Without direct observation, critical feedback is often neglected, resulting in potentially over-estimated competency in residency. Based on respondents’ comments on our proposed topic of communicating effectively with nurses, it was suggested that the topic be extended to other allied health care professionals as well, with a focus on understanding their role in the context of patient care in the ED.

Multiple suggestions were discarded on the basis that the topic(s) were abundantly addressed in existing OERs (e.g. approaches to common presentations, interpretation of ECGs) or the topic(s) were considered to exceed reasonable expectations for a clerk (e.g. ultrasound techniques, paracentesis).

## Discussion

This needs assessment survey allowed the twelve proposed topics to be stratified based on their relative importance, which will enable prioritization during the process of content creation to address these topics. Currently, our research group is in the process of creating blogs, podcasts, infographics, and videos, to address the topics that have been identified in this study. Keeping in mind that our target audience for this online educational curriculum includes students in the third and fourth year of medical school, the majority of the content creators are senior medical students and first-year residents. To ensure that the quality and execution of the content achieves a high level of excellence, our team has recruited staff EM physicians with various domains of educational expertise to supervise this process. We expect to launch this online educational curriculum on Canadiem.org within the following four to six months. 

Prior to the commencement of this investigation, we suspected from anecdotal experience that the expectations of EM educators may vary depending on a medical students’ eagerness to pursue a residency in EM. One respondent supported this suspicion by commenting that the importance of some of the core competencies proposed in the survey was partly dependent on the medical student’s inclination to pursue residency in EM. This implies higher expectations for students that are keener to pursue the specialty. However, the focus of this analysis is on the quality and content of education received by all medical students in their core EM rotation, regardless of their ambition to pursue a residency in EM. Thus, careful consideration was given to exclude topics deemed to be too advanced for medical students without intentions to specialize in EM.

Although the number of responses from program directors and clerkship directors was outweighed by staff physicians who do not hold titles, it is unlikely to affect the results of the survey, based on the assumption that the physicians who work most often with medical students are more inclined to have a superior understanding of educational needs. Although clerkship/program directors are in a unique position to affect curriculum development and possess a clear educational title, it is entirely possible that there are other physicians in their emergency department who work just as often, if not more frequently, with medical students. Thus, we did not place additional emphasis on the value of input by clerkship/program directors, compared to other EM staff physicians. Similarly, the number of staff physician responses also outweighed responses from residents. This is an expected finding, considering that at a teaching hospital, not all residents are equally involved in teaching. Generally, it is the senior residents who are more involved in teaching, and it would be reasonable to assume that the staff physicians have significantly more experience in teaching and working with medical students compared to senior residents. Thus, it is reasonable that the number of resident participants in the survey was significantly outweighed by staff physicians. 

Limitations

The data may have been subject to responder bias and the participants in the survey may not be a representative sample of all EM educators. Future investigations assessing the perceived need and importance of the nine topics that were proposed in typed free-text format in this study would be valuable. The study neglects differences in expectations and perceived needs for medical students during EM electives, particularly those aiming to specialize in the field. A future needs assessment analysis for this subgroup of students may be beneficial.

This needs assessment only solicited information about content needs, but not on needs of delivery of that content, which may be a limitation. We determined that the most effective platform (e.g. blogs, podcasts, videos, infographics) for the execution of these topics would vary according to each topic and the creativity and vision of the content creator(s). Thus, the authors of each topic may be most suitable to determine the most effective delivery method for the respective topic.

## Conclusions

We have conducted a needs assessment that has allowed us to identify a total of 21 topics that EM educators considered to be important intrinsic skills and competencies for a medical student in their EM core rotation. These topics may serve as the basis for the development of an educational curriculum for medical students who are completing their EM core rotation in clerkship. We predict that effective teaching of relevant competencies in these domains will improve the learning experiences of medical students and help them to better integrate into the EM learning environment. The next steps that follow from this needs assessment study involve literature searches to identify existing OERs for each of these topics and identify their shortcomings (if any). Subsequently, we will create content, in the form of blog posts, podcasts, and videos to better address gaps in either the quality or mode of delivery of existing content, in an effort to create comprehensive resources targeted towards medical students in their EM core rotations in clerkship.
